# A comparative study of dried apple using hot air, intermittent and continuous microwave: evaluation of kinetic parameters and physicochemical quality attributes

**DOI:** 10.1002/fsn3.241

**Published:** 2015-05-11

**Authors:** Nahid Aghilinategh, Shahin Rafiee, Abolfazl Gholikhani, Soleiman Hosseinpur, Mahmoud Omid, Seyed S. Mohtasebi, Neda Maleki

**Affiliations:** ^1^ Department of Agricultural Machinery EngineeringFaculty of Agricultural, Engineering and TechnologyUniversity of TehranKarajIran

**Keywords:** Dried apple, hot air drying, intermittent microwave, microwave drying, phenol compounds, physicochemical properties

## Abstract

In the study, the effectiveness of intermittent (IMWD) and continuous (CMWD) microwave drying and hot air drying (HAD) treatments on apple slices were compared in terms of drying kinetics (moisture diffusivity and activation energy) and critical physicochemical quality attributes (color change, rehydration ratio, bulk density, and total phenol content (TPC) of the final dried product. The temperature, microwave power, air velocity, and pulse ratio (PR) applied in the experiments were 40–80°C, 200–600 W, 0.5–2 m/s, and 2–6, respectively. Results showed that IMWD and CMWD more effective than HAD in kinetic parameters and physicochemical quality attributes. Also, results indicated CMWD had the lowest and highest drying time and effective diffusivity. The exponential model for estimating IMWD activation energy, considering absolute power (1/P) and pulse ratio were also represented. The color change in apple slices dried by HAD showed the highest change.

## Introduction

Apple is the fourth major horticultural product of human nutrition in the world. It is the pomaceous fruit of the apple tree, *Malus domestica* of the rose family of Rosaceae (Forsline et al. 2003). About 69 million tons of apples are produced in the world, half of which is produced in China. Apples are rich in terms of antioxidant. These compounds protect the body from free radical losses. Therefore, apples should be maintained for consumption in all seasons. Drying is one of the primary methods used in food preservation. The drying objective is to reduce the water to a certain level to minimize microbial waste (Akpinar and Bicer 2005). Other benefits of fruit drying are the long durability, the need for less storage space and a lighter weight for transport (Ertekin and Yaldiz 2004). Hot air drying (HAD) method is the earliest and most widely used drying method. More than 85% of industrial dryers are of convective hot air. The disadvantage of these dryers is their high‐energy consumption and lower quality. Hence, the necessity of finding fast, safe, and controllable drying methods (Kavak Akpinar et al. 2005; Motevali et al. 2011), as the application of microwave energy to food drying might be a good way to overcome the existing problems in conventional drying methods (Wang et al. 2004; Vadivambal and Jayas 2007) although the process of drying with microwaves is known to create low‐quality products with improper applications. The Influence of microwave energy inside a material is dependent on the dielectric properties that can alter the temperature distribution in the sample. In a small sample, the focusing effect of microwave as a function of time, causes overheat in the center of the sample. In such cases, the use of pulsed heating is the order of the day (Gunasekaran and Yang 2007). Akio (2012) reported that compared to hot air dryers the dried okra with microwave dryer revealed higher rehydration ratios and lower color change and bulk density. Suchismita (2012) found drying in microwave maintain the quality (color change and total phenol content) of dried Indian Borage leaves in comparison with HAD. So far, limited studies have been conducted as to intermittent microwave drying (IMWD) and the comparison among different methods of drying in terms of drying kinetics and critical physicochemical quality attributes about apple. Ergo, the objective of this study was; (1) to derive a model for estimating activation energy of intermittent microwave drying (IMWD), (2) to compare the effectiveness of intermittent (IMWD) and continuous microwave drying (CMWD) and hot air drying (HAD) treatments on apple slices in terms of drying kinetic, total phenolic content (TPC), bulk density, rehydration ratio, and color change under different conditions of drying.

## Materials and Methods

### Microwave‐convective drying system

**Figure 1:**
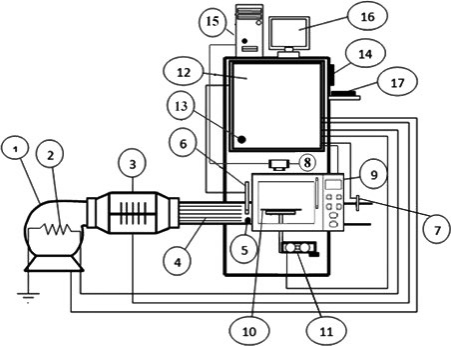
Experimental microwave‐hot air dryer. 1. fan, 2. preheating element, 3. heating elements, 4. Straightener, 5. medium velocity sensor, 6. relative humidity and temperature sensors, 7. temperature sensor, 8. digital color camera, 9. microwave oven, 10. platform, 11. load cell, 12. control unit, 13. outside temperature sensor, 14. HMI (Human–machine interface), 15. computer, 16. monitor, 17. Keyboard.

The schematic diagram of the employed microwave–convective drying system to conduct the experiments is shown in Figure 1.

The dryer consists of a microwave device, centrifugal fan (BEF‐25 = 25F4T, 6300 m^3^/h, Damandeh, Tehran, Iran), four electrical heating elements (a 750 W element in the centrifugal fan for preheating the air flow and three 2000 W elements in the air duct for heating the air flow), a control unit, a single‐point load cell, measurement sensors, and a drying chamber with one layer tray. The body of the dryer was thermally insulated with glass wool. A 2465 MHz domestic microwave oven (Samsung, OM75P; Samsung Electronics Inc., Guangzhou Daeyean Trading Company, China) with a 0–1100 W nominal power, and cavity dimensions of 300 (width) × 380 (depth) × 260 (height) mm was modified, developed, and controlled by a Programmable Logic Controller (PLC). The power supply to magnetron was controlled by a cycle controller. Integral cycle control (ON/OFF) was carried out by a time‐controller. Applied power was always 100% of its rating during ON portions of duty cycles and 0% of its rating during OFF portions. ON and OFF times are in the order of seconds. Product temperature was pulsed to reach a maximum during the ON portion and then decreased during the OFF portion (Sunjka, ). The microwave device consisted of a magnetron, an H.V. transformer, a diode and also an H.V. capacitor. Magnetron is the source of microwave energy and has either a fixed output or a variable output. Variable output magnetrons regulate the forwarded power to the processing vessel by cycling ON and OFF time. The high voltage transformer is used to turn extreme voltage current into high voltage (HV) and back. The high voltage capacitor is used for the elite battery upgrade for all the pieces of power tool. A diode is an electrical device allowing current to move through it in one direction with far greater ease than in the other. The air flow temperature was controlled with an accuracy of ±1°C using a PLC (*Programmable logic controller*), two PT‐100 temperature sensors before and after the sample tray. Outside air temperature was measured using a temperature sensor (LM35, NSC, Santa Clara, CA). In addition, relative humidity (RH) of the air flow was determined using a high‐temperature RH sensor (EE99‐03‐FP6AD 802, E+E Elektronik, Engerwitzdorf, Austria). Weight loss and moisture of the samples were measured during the drying by means of a high‐precision aluminum single‐point load cell (model 1004, Tedea‐Huntleigh, Cardiff, UK) with an accuracy of 0.001 g placed under the glass tray (diameter: 314 mm, mass: 1150 g) for a continuous measurement of the mass of the material being dried. The systems had the capability of applying several different drying treatments as follows: CMWD, IMWD, and HAD.

### Sample preparation

Apples (Red delicious variety) used in this study were supplied from a local market and then immediately stored at 6 ± 1°C until the time of the test. Prior to the drying, apples were peeled and cut, perpendicular to the fruit axis in approximately equal slices (6 mm thick). The moisture content was determined by drying the samples at 105°C in the oven for 18 h (Trabelsi and Nelson 2003). The peeled slices had the initial moisture content of about 85% wet basis (w.b.).

## Experimental procedure

There were three types of experiments where the drying regimes were as follows: (1) HAD: hot air drying was carried out at different air temperatures and air velocities ranging from 40 to 80°C and from 0.5 to 2 m/s, respectively. (2) CMWD: this drying type was done at different levels of power ranging from 200 to 600 W. (3) IMWD: a pulsing ratio (PR) was calculated for IMWD as PR = (*t*_on_ + *t*_off_)/*t*_on_, where *t*_on_ is the magnetron power on time in *s* and *t*_off_ magnetron power off time in *s*. The IMWD was pulsed at 200 to 600 W with a PR of 2 to 6. All experiments were done in triplicate.

### Effective diffusivity

Fick's second law of diffusion equation is represented in the following equation (eq. (1)):

(1) $$\frac{\partial M}{\partial t}=\nabla \left[D_{\mathrm{eff}}\left(\nabla M\right)\right].$$

Through appropriate initial and boundary conditions, Crank (Crank 1975) gave the analytical solutions to various geometries and slab objects with constant diffusivity as is given in (eq. (2)):

(2) $$\text{MR}=\frac{M_{t}}{M_{0}}=\frac{8}{\pi^{2}}\sum_{n=1}^{\infty }\frac{1}{{\left(2n-1\right)}^{2}}\exp\left(-\frac{{\left(2n-1\right)}^{2}\pi^{2}D_{\mathrm{eff}}t}{4L^{2}}\right),$$

where *D*_eff_ is the effective diffusivity (m^2^/s), L is the half thickness of the samples (*m*), *n* is a positive integer, MR is the moisture ratio, *M*_t_ is the moisture content at a specific time (g water/g dry base), *M*_o_ is the initial moisture content (g water/g dry basis).

In this study, to estimate the water effective diffusion coefficient more accurately, we used the first three terms of the series in the analytical solution of the Fickian diffusion model (eq (1)). The effective moisture diffusivities are typically determined by plotting experimental drying data in terms of ln (MR) versus time.

### Bulk density measurement

The bulk density of samples was determined by weighing the samples and then placing them in a container with a determined volume, filled with toluene (Baysal et al. 2003). The volume expansion in the container was recorded at 25°C. The bulk density was determined via the ratio of the samples weight to the volume expanded in cubic meters.

### Determination of total phenolic content

A modified method was used to analyze the total phenolic content (TPC) (as gallic acid equivalent (GAE)) using the Folin–Ciocalteu (FC) (Vega‐Gálvez et al. 2009). Dried apple (2.00 g), which had been crushed to powder using a Waring blender, was mixed with 100 mL cool water at room temperature and constantly swirled with an orbital shaker for 1 h. The extracts were filtered (Whatman filter paper nr 1) in a Buchner funnel. A 0.5 mL aliquot of the apple extract was transferred to a glass tube containing 0.5 mL FC reagent, vortex‐mixed and kept for 5 min. Next, 2 mL of 20% Na_2_CO_3_ was introduced and incubated for 15 min at ambient temperature. After adding 10 mL of ultra‐pure water, the formed precipitates were removed in 5 min by centrifugation. Finally, absorbance was measured at 725 nm with a spectrophotometer (Spectronic‐20 Genesys, IL, USA) and compared to a GA calibration curve. Results were expressed as mg GA/100 g dry matter. All reagents were purchased from Merck chemical company (Merck KGaA, Darmstadt, Germany), and all measurements were carried out in triplicate.

### Determination of rehydration ratio

This parameter was determined by immersing dried samples in distilled water at specified rehydration temperatures (20°C) for 14 h (Lewicki 1998). The water was drained and the slices weighed with 2 h intervals. Rehydration ratio was defined as the rehydrated samples ratio of weight (*W*_r_) to the dry weight (*W*_d_) of the sample (eq. (3)):

(3) $$RH=\frac{W_{r}}{W_{d}}\times 100.$$

### Color change measurement

The amount of color change is usually taken into consideration when comparing the color changes in food drying (Maskan 2000). Sample color was measured before and after drying by a Hunter Lab ColorFlex, A60‐1010‐615 model colormeter (HunterLab., Reston, VA) at four different points on the apple's surface for all of experiments. The color values were indicated as L* (whiteness/darkness), a* (redness/greenness), and b*(yellowness/blueness). Also, the color change was calculated from the L*, a*, and b* values', using eq. (4) and it was used to describe the color change during drying:

(4) $$\Delta E=\sqrt{{\left(L^{*}-L_{{}_{0}}^{*}\right)}^{2}+{\left(a^{*}-a_{{}_{0}}^{*}\right)}^{2}+{\left(b^{*}-b_{{}_{0}}^{*}\right)}^{2}},$$

where L*, a*, and b* indicate the brightness, redness, and yellowness of dried samples respectively and subscript “0'' refers to the color reading of fresh apple (Maskan 2000).

### Statistical analysis

Analysis of variance (ANOVA) was conducted by a factorial test in a complete randomized design with three replicates in HAD and IMWD and complete randomized design with three replicates in CMWD, so as to evaluate the individual effect of independent variables on the effective diffusivity, rehydration ratio, bulk density, color change, and total phenolic content. All the data were analyzed using SAS 9.1 software package (SAS Institute Inc., Cary, NC). This means that the comparisons were drawn using Fisher's protected least significant difference (LSD) at a probability level of 5%.

## Result and Discussion

### Drying kinetic

**Figure 2:**
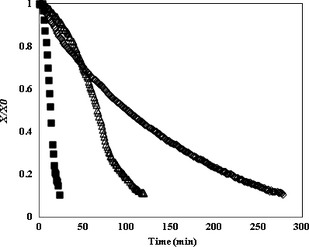
Typical drying curves for apple slices dried with hot air drying (◊), continuous microwave drying (■), and intermittent microwave drying (∆).

**Figure 3:**
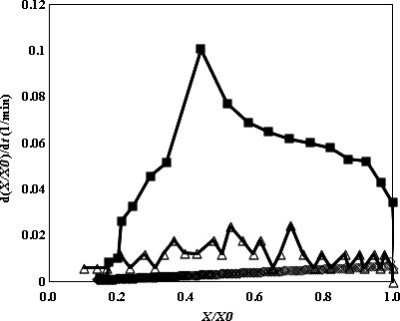
Typical drying rate curves for apple slices dried with hot air drying (◊), continuous microwave drying (■), and intermittent microwave drying (∆).

The typical drying curves of apple slices dried by various methods are shown in Figure 2. Dimensionless moisture content (*X*/*X*_0_) of the samples continuously decreased with the increase in the drying time. It is evident from the examination of these curves that the drying of apple by CMWD treatment was much faster than by HAD or IMWD. For instance, it took 23, 120, and 278 min to reach an *X*/*X*_0_ of about 0.1, for CMWD, IMWD and HAD, respectively. The drying rates of apple slices dried by various methods are given in Figure 3. It is obvious that no constant rate period was observed during drying due to the hygroscopic nature of these materials (Fig. 3). Generally, the curves had an initial warming‐up period followed by a falling rate period (Van Arsdel 1973). The drying rate increased during the early stages of CMWD. At the initial phase of drying, due to the high moisture content, the amount of dipole molecules was high, resulting in a high microwave energy absorption by the product; the absorbed microwave energy was then converted into thermal energy leading to a rapid temperature increase within the product (McLoughlin et al. 2003). A falling rate period was also observed for the CMWD, following the maximum drying rate. Similar results were obtained for CMWD of potato (Wang et al. 2004), banana (Maskan 2000), leek (Dadali and Özbek 2008), garlic (Sharma and Prasad 2001), and parsley (Soysal et al. 2006). For the IMWD treatment, the drying rate increased in the beginning, followed by a fall in each working cycle (*t*_on_ + *t*_off_) of microwave. Since microwave power off time provided a rest time for moisture and temperature redistribution within the product and microwave power on time created the warming‐up period (Beaudry et al. 2003). It is obvious from Figure 3 that due to the initial heating up of the products, the drying rate for HAD have an ascending trend in the primary stages of drying than a descending trend toward the end of the drying process due to the second drying step (falling rate period) was observed. Similar findings have been reported by Yadollahinia et al. (2009) and Batista et al. (2007) for potato and chitosan, respectively.

### Calculation of the effective moisture diffusivity

The range of moisture diffusivities in CMWD, IMWD and HAD varied from 1.92 × 10^−8^ to 1.58 × 10^−7^ m^2^/s, from 1.26 × 10^−8^ to 4.94 × 10^−8^ m^2^/s and from 7.54 × 10^−9^ to 2.86 × 10^−8^ m^2^/s, respectively (Tables 1 and 2). The CMWD and HAD processes had a highest and lowest effective moisture diffusivity in the dried apple slices, because CMWD increases temperature and consequently the water vapor pressure further than IMWD and HAD, that helps in faster diffusion of moisture toward the surface. The highest and lowest effective moisture diffusivities of apple slices in CMWD were found at 600 and 200 W, respectively. Additionally, the maximum values for effective moisture diffusivities of apple slices in IMWD and HAD treatments were found at 600 W, PR of 2, a temperature of 80°C, and an air velocity of 2 m/s. As expected, effective moisture diffusivity significantly increased with the increase in the drying temperature, air velocity, and microwave power due to the faster diffusion of moisture toward the surface, while effective moisture diffusivity decreased with the rise in the PR. Similar results were obtained for drying of tomato (Sacilik et al. 2006), pear (Nguyen et al. 2006), durian (Jamradloedluk et al. 2007), banana (Nguyen and Price 2007), and plum (Goyal et al. 2007).

**Table 1:** Physicochemical quality attributes and effective diffusivity for apple slices dried by continuous microwave drying (CMWD) and intermittent microwave drying (IMWD)^a^

Treatment	Factor level	Rehydration ratio	Color change	TPC (mg GA/100 g)	Bulk density (g/m^3^)	Deff (m^2^/s)
CMWD	Power					
	200	2.599^c^	2.374^c^	171.396^c^	0.767^a^	1.92 × 10^−8c^
	400	2.952^b^	3.764^b^	239.630^b^	0.544^b^	1.04 × 10^−7b^
	600	3.450^a^	5.155^a^	331.652^a^	0.480^b^	1.58 × 10^−7a^
IMWD	Power					
	200	4.065^b^	3.055^c^	99.063^c^	0.568^a^	1.26 × 10^−8a^
	400	4.333^a^	6.556^b^	122.524^b^	0.518a^b^	1.63 × 10^−8a^
	600	4.537^a^	7.268^a^	145.984^a^	0.427^c^	2 × 10^−8a^
	PR					
	2	4.168^a^	4.620^b^	213.288^a^	0.502^b^	4.94 × 10^−8a^
	3	3.740^ab^	6.023^ab^	171.729^b^	0.561^b^	4.27 × 10^−8a^
	6	3.129^b^	7.412^a^	94.695^c^	0.467^a^	3.24 × 10^−8a^

a The means with minimum common letter in same column for each factor are not significantly different (*P < *0.05) according to LSD's multiple range test.

**Table 2:** Physicochemical quality attributes and effective diffusivity for apple slices dried by hot air drying^a^

Factor level	Rehydration ratio	Color change	TPC (mg GA/100 g)	Bulk density (g/m^3^)	*D*_eff_ (m^2^/s)
Temperature (°C)					
40	2.001^d^	3.811^d^	101.349^a^	0.858^a^	9.47 × 10^−9a^
50	2.383^cd^	4.238^d^	77.832^b^	0.797^b^	1.07 × 10^−8a^
60	2.743^bc^	5.672^c^	60.385^c^	0.726^c^	1.48 × 10^−8a^
70	3.014^ab^	7.449^b^	44.276^d^	0.681^cd^	1.83 × 10^−8a^
80	3.282^a^	8.825^a^	25.082^e^	0.631^d^	2.86 × 10^−8a^
Air velocity (m/s)					
0.5	2.461^b^	7.325^a^	53.677^c^	0.804^a^	7.54 × 10^−9a^
1	2.715^ab^	5.768^b^	62.288^ab^	0.743^b^	1.44 × 10^−8a^
2	2.998^a^	4.903^c^	69.390^a^	0.669^c^	2.71 × 10^−8a^

a The means with minimum common letter in same column for each factor are not significantly different (*P < *0.05) according to LSD's multiple range test.

### Estimation of activation energy

In this study, two independent variables were used in the drying process throughout IMWD treatment. The Arrhenius equation was used in a modified form to illustrate the relationship between the kinetic rate constant, while independent variables were employed in calculating the activation energy (Table 3). After data evaluation, the dependence of the kinetic rate constant on the absolute power (1/P) and the PR were represented with one exponential equation for IMWD (eq. (5)). Equations (6) and (7) were used for HAD and CMWD in which the activation energy was determined from the slope of the Arrhenius plot ln (*D*_eff_) versus 1/T and 1/P, respectively.

**Table 3:** Activation energy values for intermittent microwave drying (IMWD), continuous microwave drying (CMWD) and hot air drying (HAD)

Drying method	Air velocity (m/s)	*E*_a_
IMWD	–	4.93 (W/g)
CMWD	–	4.14 (W/g)
	0.5	25.23 (kJ/mol)
HAD	1	23.58 (kJ/mol)
	2	21.26 (kJ/mol)

(5) $$D_{\mathrm{eff}}=2.26\times 10^{-7}{\text{PR}}^{-0.604}\exp\left(-\frac{4014m}{P}\right)$$

(6) $$D_{\mathrm{eff}}=D_{0}\exp\left(-\frac{E_{a}}{{\text{RT}}_{a}}\right),$$

(7) $$D_{\mathrm{eff}}=D_{0}\exp\left(-\frac{E_{a}m}{P}\right),$$

where *E*_a_ is the activation energy (W/g), m is the mass of raw sample (g), *D*_0_ is the pre‐exponential factor (m^2^/s), P is the power (W), *T*_a_ is temperature of air (°C), and *R* is the universal gas constant (kJ/mol K).

The fitted model, account for the 89% of the variability in *D*_eff_ at the 99% confidence level. According to the constructed models, in order to facilitate the diffusion of moisture from apple slices, CMWD requires the lowest activation energy, compared with IMWD (Table 3) and HAD requires the lowest activatin energy at 2 m/s velocity since the rate of the nonbond and bond water removal is the highest in CMWD and HAD at 2 m/s velocity.

### Total phenolic content

The TPC significantly increased with the increase in the microwave power and air velocity (*P *<* *0.01), but it sharply (*P *<* *0.01) fell with the increase in the temperature and PR (Tables 1 and 2). However, due to its simplicity and rapidity, microwave treatment could be effective in releasing antioxidant compounds from agricultural byproducts. Long process times ultimately lead to a considerable reduction in nutrient property and antioxidant activity (López et al. 2010). It is also mentioned that the long drying time of product placement in the atmospheric oxygen can have such adverse effects on the quality as reduction in the amount of phenolic compounds (Aghae et al. 2013). Moreover, exposure to high temperatures disrupts cells and may also result in the release of oxidative and hydrolytic enzymes. These enzymes are capable of oxidizing phenolic compounds. The highest and lowest values of TPC of apple slices in IMWD was observed in microwave power of 600 W, PR of 2 and 200 W, and PR of 6, respectively. The CMWD and HAD rendered the highest and lowest TPC in dried apple slices.

### Bulk density

As already provoked, the bulk density significantly decreased (*P* < 0.01) with the increase in the microwave power, temperature and air velocity, whereas it significantly fell (*P *<* *0.01) with the rise in PR (Tables 1 and 2). The decreasing trend of the bulk density with an increase in temperature and microwave power is in agreement with the findings of previous researchers (Khraisheh et al. 2004; Pimpaporn et al. 2007; Thuwapanichayanan et al. 2011). This might be due to the change in sample texture during drying process, an increase in the microwave power and temperature causes a drop in the apparent and total densities and a slight increase in the total porosity, also this is because of the fact that drying time at low temperature, velocity and power is higher than that at high temperature, air velocity, and power (Nathakaranakule et al. 2010) and this probably influenced the density, structure, and porosity of the obtained dried apples (Kaur and Singh 2014). The increasing trend of the bulk density with an increase in PR, might be due to the declining radiation time of the microwave. The mean values of apple slices bulk density in HAD, CMWD, and IMWD varied from 0.63 to 0.85 g/cm^3^, from 0.48 to 0.76 g/cm^3^ and from 0.42 to 0.56 g/cm^3^. The results also indicated that the bulk density of the samples dried by IMWD and CMWD methods were lower than in the sample dried by HAD. During HAD, the apparent density increases, while porosity of the final product decreases due to solids gain (Krokida and Maroulis 1997). It is clear that IMWD had the lowest bulk density. This improvement possibly can be attributed to the temperature and moisture redistribution during the tempering period. The tempering period helps to reduce the temperature and moisture gradient, resulting in a decrease in the internal stress.

### Rehydratio ratio

The dried product rehydration characteristic can be used as a quality indicator. Rehydration is a complex process and indicates the physicochemical changes caused by drying (Lewicki 1998). The rehydration ratio significantly increased with the increase in air temperature, air velocity and microwave power while it decreased with the surge in PR (*P* < 0.01, Tables 1 and 2). This might be due to a suitable change in sample texture during microwave and hot air drying at high temperature, air velocity and microwave power (Kaur and Singh 2014). IMWD rendered the highest rehydration ratio, followed by CMWD and HAD. According to Sumnu et al. (2005), the high internal pressure produced by microwave drying can cause expansion and puffing of the material and thus reduce the density of the structure. This less dense structure has a higher capacity to absorb water and reconstitute.

### Color change

It was observed that IMWD and CMWD methods at the lowest power and PR and HAD at the lowest temperature entailed less color change compared to other treatments (Tables 1 and 2). This trend may be due to the reduction in the Maillard reaction occurring at high air temperatures and microwave power levels (Kaur and Singh 2014). The data also pointed that the color of the samples dried by IMWD and CMWD methods showed lower color change than the samples dried by HAD. According to Schiffmann (1995), microwave energy transfers liquid into surface of material, and this liquid is usually converted into vapor. Therefore, the microwave drying process does not create the phenomenon of surface overheating, makes less color change in the surface. It is obvious that CMWD retained the highest color quality of the fresh apple slices. The lower color degradation of CMWD may be due to the reduced drying time. The degree of color change was significantly dependent on the drying temperature, PR, air velocity and microwave power (Tables 1 and 2). A significant increase in color change value could happen with an increase in the temperature and microwave power. Ultimately, the amount of color change was more at a higher PR, a fact similar to the results presented by Esturk (2012).

## Conclusion

In the present study, kinetic parameters and quality characteristics of IMWD, CMWD, and HAD of apple slices were compared. The drying rate curve in each of three drying methods (CMWD, IMWD and HAD), had an initial warming‐up period followed by a falling rate period. The result indicated IMWD and CMWD more effective than HAD in kinetic parameters and physicochemical quality attributes. CMWD had a faster drying trend in comparison with other studied methods. The drying time of IMCD was about three times longer than CMWD. The mean values of activation energy in HAD, IMWD, and CMWD obtained 21.23 to 25.26 kJ/mol, 4.93 W/g and 4.14 W/g, respectively. The highest values of effective diffusivity between the three drying methods (CMWD, IMWD and HAD) were found in CMWD. Furthermore, CMWD rendered the lowest color change and the highest TPC, while IMWD had the lowest and highest bulk density and rehydration ratio. Therefore, CMWD is recommended for the drying of apple slices when the TPC and the amount of color change are at stake.

## Conflict of Interest

None declared.
